# Adenosine deaminases that act on RNA, then and now

**DOI:** 10.1261/rna.079990.124

**Published:** 2024-05

**Authors:** Brenda L. Bass

**Affiliations:** Department of Biochemistry, University of Utah, Salt Lake City, Utah 84112, USA

**Keywords:** ADAR, RNA editing, dsRNA, inosine, innate immunity

## Abstract

In this article, I recount my memories of key experiments that led to my entry into the RNA editing/modification field. I highlight initial observations made by the pioneers in the ADAR field, and how they fit into our current understanding of this family of enzymes. I discuss early mysteries that have now been solved, as well as those that still linger. Finally, I discuss important, outstanding questions and acknowledge my hope for the future of the RNA editing/modification field.

## INTRODUCTION

It was a melancholy, rainy night in Seattle and to cheer myself up, I made plans to go to a movie with my friend Pat Kato. Pat remembers a Dennis Quaid movie—I have no idea. I do remember that after the movie we were talking in the lobby as we said our goodbyes, and I told Pat that I was trying to decide if I should go back to the lab to develop the autoradiogram of my thin-layer-chromatography (TLC) plate. I felt like things were going so, so, slow in the lab. I envisioned that the word FAILURE that was already flashing in capital letters behind my eyes would soon make it to my forehead, and I would never get a job.

Would developing the autoradiogram cheer me up or lower my spirits further? Always the optimist, I headed to the lab. About a year and a half into my postdoc, in trying to understand why antisense RNA techniques did not work to repress gene expression in *Xenopus laevis* embryos, as a control, I injected a double-stranded RNA (dsRNA) into a recently fertilized embryo and discovered what appeared at first to be an unwinding activity (Bass and Weintraub 1987), but more recent experiments suggested was an activity that covalently modified dsRNA. A conversation riding up a ski lift with my former mentor Tom Cech, and our friend Olke Uhlenbeck, had led to the idea that the best next step would be to make four dsRNAs, each with a different ^32^P-labeled nucleotide. We reasoned that if one particular nucleotide was subject to modification by the activity, after the dsRNA was digested to nucleotides, the product would be labeled, and this would reveal which nucleotide(s) had changed.

**FIGURE 1.**
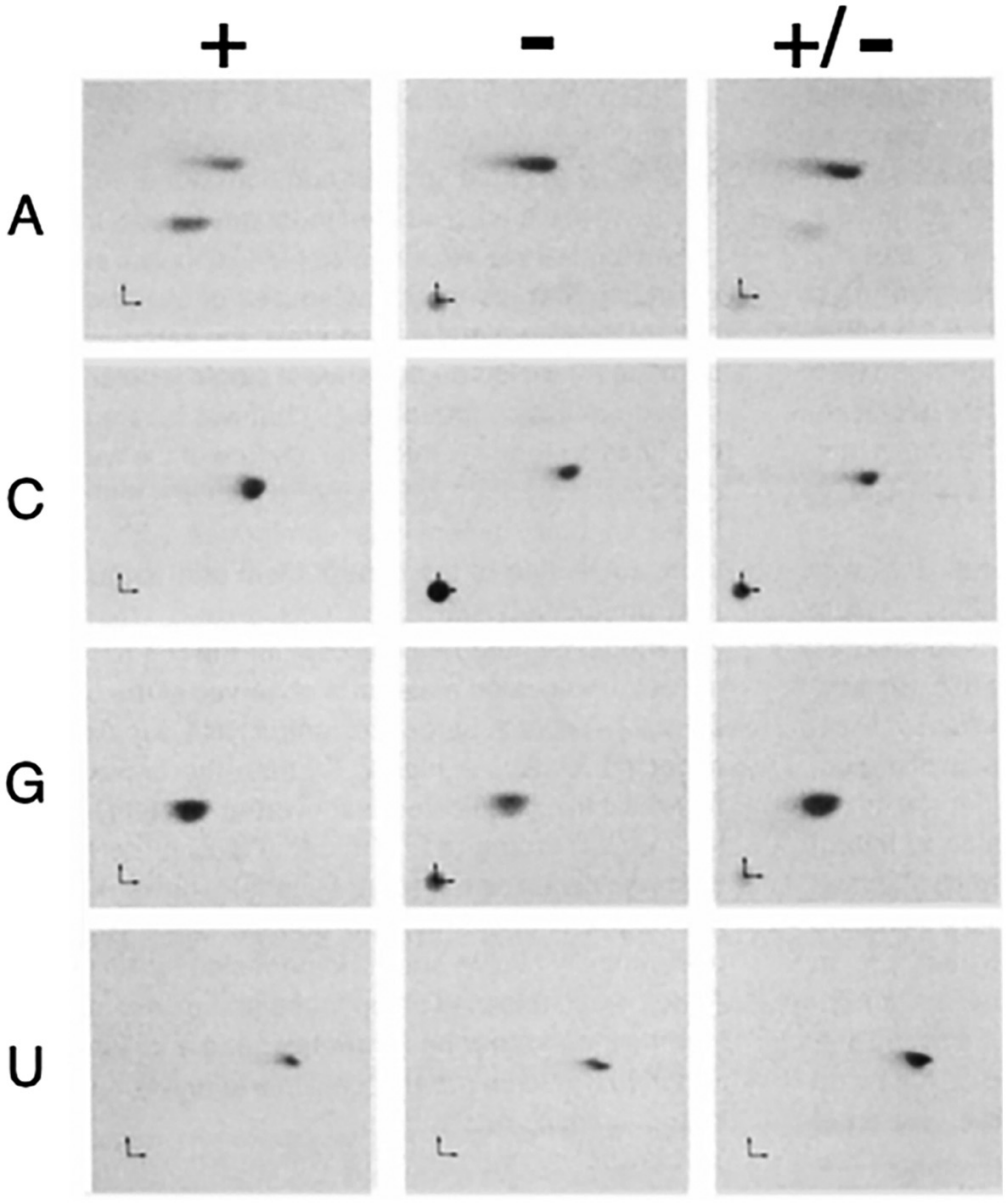
TLC analyses reveal adenosine-to-inosine (A-to-I) editing. Two-dimensional TLC analyses using four different dsRNAs, each containing one strand that was labeled during in vitro transcription with ^32^P-ATP (A), ^32^P-CTP (C), ^32^P-GTP (G), or ^32^P-UTP (U). dsRNAs were reacted for 1 h in a *X. laevis* embryo nuclear extract (+), or a heat-inactivated embryo nuclear extract (−). After incubation, reactions were deproteinized, digested to nucleotides with nuclease Pl, and each sample, as well as a mixture of both (+/−), were spotted at the *bottom left-hand* corner of a cellulose chromatography plate. Directions of chromatography are indicated by small arrows at the plate origins, with the first dimension indicated by the *vertical* arrow and the second dimension indicated by the *horizontal* arrow. Additional details and original figure are in Bass and Weintraub (1988).

After flying home from the meeting, I immediately did the experiment, separated the radiolabeled nucleotide products on two-dimensional TLC plates, and put a piece of film on top of the plate to capture the result; THIS was the autoradiogram I was going back to the lab to develop. And there it was. A new spot in the ^32^P-A-labeled dsRNA (Fig. 1; Bass and Weintraub 1988), and one that, from my graduate studies (Bass and Cech 1984), I was almost certain was inosine. It was after midnight, and not even the custodians were around, and I circled the floors until I found my friend Jim Roberts. He listened patiently, although in my opinion not nearly as excited as he should have been, and finally said, “Gee, that's great, B!”

As scientists we are lucky to get even one true discovery in our career, and those that are lucky know the exhilaration of thinking for a moment that you know something no one else does. But even back then when things were less competitive, elsewhere in the world, others were making similar discoveries. I was not alone in my first glimpse of ADAR as an unwinding activity in *X. laevis* (Rebagliati and Melton 1987), and Richard Wagner and Kazuko Nishikura were busy detecting ADARs in mammalian cells (Wagner and Nishikura 1988; Wagner et al. 1989). Ensuing years brought the first purification and cloning of the enzyme (Hough and Bass 1994; Kim et al. 1994a,b; O'Connell and Keller 1994; O'Connell et al. 1995), followed by cloning of the enzyme from many animals, the realization that ADARs were a family of enzymes (for review, see Bass 2002), and a slew of papers that no one cites anymore because they are not aware that ADARs were initially called dsRAD or DRADA (Bass et al. 1997).

After my meaningful night in the lab, I quickly realized that I was now part of a relatively new field called RNA editing, initiated with Rob Benne's discovery of nonencoded nucleotides in a mitochondrion-encoded mRNA of a kinetoplastid protozoan, or trypanosome (Benne et al. 1986), and followed closely by the first example of editing in a nuclear-encoded RNA, the mRNA for apolipoprotein B (Chen et al. 1987; Powell et al. 1987). Soon, major meetings started to include a session on RNA editing, typically the very last session, and I frequently saw Ken Stuart, Larry Simpson, Donna Driscoll, Jonatha Gott, Harold Smith, and others. Here we are almost 40 years later, and I am awed by the state of the field and the discoveries that have pushed the field forward. In the sections below, I discuss the mysterious observations made early on in studies of ADARs that have now been solved, emphasize those that still linger, and detail my hope for the future of the RNA modification/editing field as a whole. For those wishing for an in-depth and detailed background of the ADAR RNA editing field, many excellent and recent reviews exist (Nishikura 2016; Erdmann et al. 2021; Herbert 2021; Pfaller et al. 2021; Quin et al. 2021; Rosenthal and Eisenberg 2023). I also point to reviews in this issue by colleagues in the ADAR field, Peter Beal and Jin Billy Li.

### Where are the accessory factors?

#### Sequence preferences

Early studies of ADARs using *Xenopus* eggs (Kimelman and Kirschner 1989) or their extracts (Wagner et al. 1989) noted adenosine-to-inosine (A-to-I) changes preferentially at sites with certain 5′ or 3′ nearest neighbors, with the most striking feature being a preference for anything but guanosine 5′ of the target adenosine. More systematic studies using extracts and purified proteins refined the nearest-neighbor preferences (Polson and Bass 1994), and ultimately, by incorporating quantitation of Sanger sequencing data and highly purified enzymes, human ADAR1 was established as having 5′ preferences of U > A > C > G and 3′ preferences of G > C≈A > U. ADAR2 was found to have similar, but distinct, preferences (5′ U > A > C > G; 3′ G > C > U ≈ A). The latter study found nearly identical preferences using just the deaminase domains alone, consistent with prior domain swapping experiments (Wong et al. 2001), and more recent studies showing that swapping a “5′ binding loop” within the deaminase domain of human ADAR2 into human ADAR1 changes its substrate preferences to those of ADAR2 (Wang et al. 2018).

Initially it was assumed that preferences would be conferred by accessory factors, but once ADARs were cloned, it was realized that the purified enzymes could re-create the preferences (Polson and Bass 1994). Further, mutagenesis experiments interrogating editing at the UAG stop codon in hepatitis delta virus (HDV) (Polson et al. 1996) showed that the sequence preferences observed in vitro with purified protein were identical to those observed in vivo. Indeed, as ADARs were studied in other organisms (Morse and Bass 1999), it became clear that preferences were a conserved and intrinsic property of the enzyme family. Soon after ADARs were discovered, Rich Roberts and colleagues discovered that the mechanism of the *M.HhaI* DNA cytosine-5 methyltransferase involved flipping the target cytosine out of the DNA helix and into the catalytic active site (Klimasauskas et al. 1994; Roberts 1995). By analogy, it was proposed that ADAR enzymes would involve a “base-flipping” mechanism (Hough and Bass 1997). Indeed, subsequent biochemistry experiments suggested that guanosine was disfavored 5′ of an ADAR targeted site because the purine stacking made it more difficult for flipping to occur (Kuttan and Bass 2012). Ultimately, structural studies elegantly validated a base-flipping mechanism (Matthews et al. 2016) and also documented other important interactions that contribute to ADAR preferences (Thomas and Beal 2017).

What was *not* immediately apparent in the early studies was just how important knowledge of ADAR preferences would be to gaining confidence during mapping of ADAR editing sites within endogenous transcripts. In situations where reads are low, a matched genomic sequence is unavailable, or animals/cells lacking ADAR genes are difficult to acquire, it is hard to gain confidence that an A-to-G change in a cDNA derives from an A-to-I change in the RNA, rather than a single-nucleotide polymorphism or sequencing error. In these cases, the observation that very few transitions have a 5′ G gives one courage to go forward.

#### Selectivity

In addition to observations of nearest-neighbor preferences, early studies using extracts from *Xenopus* eggs or mammalian cultured cells also found that perfectly paired dsRNAs longer than ∼50 bp were deaminated promiscuously, with 40%–50% conversion of adenosines to inosines at reaction completion (Bass and Weintraub 1988; Wagner et al. 1989; Polson and Bass 1994). However, with shorter duplexes the reaction reached completion at a lower percent deamination (Nishikura et al. 1991). During the ADAR reaction, AU base pairs within a dsRNA substrate are changed to IU mismatches, and consistent with this, early studies showed that as the reaction proceeded, the RNA became more sensitive to single-strand specific ribonucleases (Bass and Weintraub 1988; Wagner et al. 1989). Because of the increase in mismatches as the ADAR reaction proceeds, the dsRNA substrate becomes less thermodynamically stable, and while it is rare that two strands of a dsRNA actually separate after deamination, it is this property that led to the initial characterization of ADARs as an “unwinding” activity (Bass and Weintraub 1987; Rebagliati and Melton 1987; Wagner and Nishikura 1988). Since the reaction stopped before all adenosines were deaminated, it was proposed that the reaction reached completion when the dsRNA was too single-stranded to be recognized as dsRNA; this process, whereby only a subset of the preferred adenosines are deaminated, is called “selectivity” (Bass 1997). Recent high-throughput analyses, discussed in more detail below, corroborate the idea that progressive disruption of a completely base-paired A-form double helical structure leads to lower editing levels (Zambrano-Mila et al. 2023).

The first naturally occurring ADAR substrates identified were viral transcripts (Cattaneo et al. 1988; Bass et al. 1989), and consistent with observations made in vitro using long, synthetic dsRNAs, cDNAs derived from these substrates typically showed ∼50% A-to-G changes (Bass 1997), a phenomenon called “hypermutation” at the time, and now often referred to as hyperediting (Porath et al. 2017). Today, ADARs are known to target many viruses (for review, see Pfaller et al. 2021), and many show the “hypermutation” or “hyperediting” type of modification. Importantly, when these viral transcripts were first being characterized, editing sites were typically revealed by sequencing cloned cDNAs, thus giving an accurate representation of the editing events in a single molecule (e.g., Kumar and Carmichael 1997). The latter is not always possible with more modern protocols, for example, when qRT-PCR is used to amplify a population of edited transcripts, or when editing events are compiled from shorter RNA-seq reads. Such protocols give an ensemble of the editing sites that exist in the many different molecules within the sample, which may or may not exist together in a single transcript; this highlights the advantage of single-molecule sequencing protocols like nanopore for mapping editing sites. Regardless, when endogenous cellular substrates were identified, and their inosines mapped either in cloned cDNAs or PCR-amplified from mixed populations, it was clear that it was rare to find 50% of the adenosines in a single molecule deaminated (Morse and Bass 1999; Morse et al. 2002). Again, it was assumed that the discovery of accessory proteins that allowed selectivity was forthcoming.

A clear difference between the viral and cellular transcripts was their predicted dsRNA structures. Editing of viral transcripts was presumed to occur within completely base-paired dsRNA derived from viral replication intermediates, while sequences of cellular substrates showed predicted structures that were largely double-stranded, but periodically interrupted by mismatches, bulges, and loops. A study in the late 1990s showed that inserting internal loops ≥6 nt into a completely base-paired dsRNA could dramatically decrease the number of adenosines deaminated at complete reaction (Lehmann and Bass 1999). By comparing a variety of different dsRNAs, the authors concluded that internal loops were recognized as the end of a dsRNA by an ADAR, and thus could turn a long dsRNA into a series of shorter ones that would be deaminated more selectively. The importance of structural disruptions to selectivity was emphasized by a phylogenetic analysis of the R/G editing site that occurs in certain glutamate receptors (Aruscavage and Bass 2000). It was observed that the structures that encompass the editing site show a different type of sequence conservation than commonly observed in tRNAs and rRNAs. For the latter, phylogenetic analyses show compensatory mutations in base-paired regions, emphasizing that it is the dsRNA structure, rather than its sequence, that is important for function. In contrast, but not surprising given the importance of structural disruptions to selective editing, for the hairpin that contains the R/G editing site, the bases in unpaired regions (e.g., mismatches and hairpin loop) varied so as to maintain an unpaired state, while the base pairs, even those involving intronic sequences, were conserved at the sequence level. Phylogenetic analyses in arthropods also emphasized these trends and again pointed to the importance of RNA structure for ADAR editing (Reenan 2005).

### A model for how structural disruptions affect ADAR editing

But what about the complicated array of mismatches, bulges, and loops that occur in many cellular substrates, for example, the inverted Alus that are so often targeted by ADARs in human cells (Schaffer and Levanon 2021)? How do these structural disruptions affect ADAR editing? While answering this question seems daunting, recent studies using a massively parallel synthetic approach provide a clear advance in our understanding (Uzonyi et al. 2021; Zambrano-Mila et al. 2023).

Both studies center on two RNA hairpins of 146 bp with a 46-nt loop, one based on a short interspersed nuclear element (SINE), the mouse B2 repetitive element, and the second based on the 3′ untranslated region of the fluorescent reporter mNeonGreen. About 2000 sequence variants that included random structural disruptions, as well as systematically positioned mismatches, bulges, and changes to the stem length, were synthesized. The oligo libraries were transfected into HEK293T cells, allowing for editing from endogenous human ADAR1, or alternatively, experiments were designed so that ADAR expression only occurred via plasmids encoding either human ADAR1 or ADAR2. Combining the data from both studies, the take-home lesson is that structural disruptions correlate with an increase in editing at an adenosine a defined distance away: ∼35 bp 5′ of the structural disruption for ADAR1, and ∼26 bp 5′ of the structural disruption for ADAR2. Domain swapping experiments indicate that the identity of the dsRNA binding domains (dsRBDs), although not their number, dictates the effect of the structural disruption and its characterized offset of 35 or 26 nt.

To validate their outcomes, the authors surveyed endogenous transcripts containing SINE elements, in particular inverted Alu elements in human transcripts, focusing on those where the dsRNA structure could be predicted with the most confidence. They analyzed the correlation between levels of editing at specific sites and structural disruptions, and consistent with the fact that most Alu elements are edited by ADAR1, these studies reiterated the 35-nt offset observed with the synthetic hairpins. The authors also performed experiments where they created structural disruptions in defined targets to see if they could boost editing, as might be warranted when using ADAR editing for therapeutic purposes (Pfeiffer and Stafforst 2023). While these experiments worked in some cases, the effects were small, and in other cases editing levels did not change. This emphasizes that while important progress has been made, we still have a lot to learn about the rules of ADAR editing.

**FIGURE 2.**
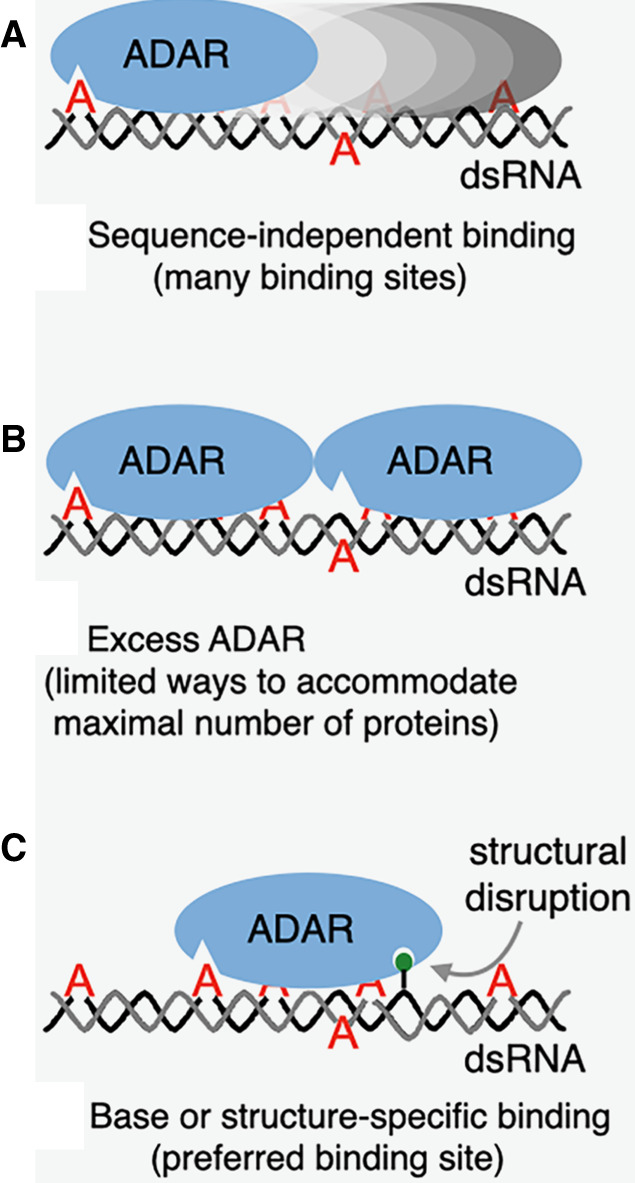
Model for effects of structural disruptions on ADAR editing. Oval-shaped ADAR enzymes are shown, with a triangular binding site for target adenosines (red). For the sake of illustration, each ADAR is assumed to interact with ∼40 bp of an ∼90 base-paired dsRNA (complementary gray and black strands). (*A*) Because ADARs are not sequence-specific, many binding sites exist, which theoretically equal: (dsRNA length in base pairs) − (binding site size in base pairs) + 1, or 90 − 40 + 1 = 51. (*B*) In the presence of excess ADAR there will be one most energetically favorable complex, in this hypothetical example, two ADAR proteins per dsRNA, which can only be accommodated in one way, thus limiting the adenosines that can be targeted. (*C*) Under conditions of limiting ADAR, a structural disruption (green lollipop) is recognized so that ADARs bind in a particular register, creating an editing hotspot, and again, limiting the adenosines that can be targeted.

That said, the studies highlight many properties of dsRNA binding proteins (dsRBPs) (Tian et al. 2004; Cottrell et al. 2024), and Figure 2 illustrates a simple model to explain the observed offset. The model starts with Pete von Hippel's elegant treatise of how sequence-independent binding proteins, like ADARs, interact with a “lattice” of binding sites, for example, a nucleic acid double-helix (McGhee and von Hippel 1974; see Discussion in Parker et al. 2006). While some studies suggest a large, 50 bp footprint for ADARs (Song et al. 2020), clearly ADARs can edit shorter duplexes, and further studies seem warranted. Regardless, for the sake of this discussion, if one assumes a footprint of 40 bp, a completely base-paired dsRNA of 90 bp will present 90 − 40 + 1, or 51 binding sites (Fig. 2A; McGhee and von Hippel 1974). Assuming that all binding sites are equally probable, it seems logical that the observed editing sites would be preferentially targeted based on their nearest-neighbor preferences. Extending the model to the condition of a large excess of protein, at equilibrium the thermodynamically most stable ADAR•dsRNA complex would be the one that maximizes the number of interactions, which would be dictated by how many proteins would fit on the RNA; for the purposes of discussion, the model shows two (Fig. 2B). In this case it is assumed there is only one way to fit two proteins on the RNA, so editing would show hotspots if the binding sites positioned an ADAR over an adenosine in good sequence context; however, in contrast to the scenario in Figure 2A, there are fewer binding sites, and thus, fewer adenosines are edited.

Just as saturating concentrations of ADARs would restrict the number of ways that ADARs could bind to dsRNA, if a structural disruption allowed a sequence-specific interaction, and thus, a preferred, high-affinity binding site, it would dictate a binding register (Fig. 2C). Editing would be enhanced at a specific adenosine within the binding site, but many sequence-independent sites would be occluded, limiting additional editing sites. Indeed, the recent studies show that structural disruptions increase editing of an adenosine target at a distance, while blocking editing in the intervening region (Uzonyi et al. 2021; Zambrano-Mila et al. 2023).

Is it likely that structural disruptions allow sequence-specific interactions? In a right-handed double-helix, either a B-form dsDNA or an A-form dsRNA, most of the functional groups that enable sequence-specific interactions are in the major groove, and there are many examples of proteins that make sequence-specific interactions in the major groove of B-form dsDNA (Rohs et al. 2010). However, an A-form helix has a much deeper and more narrow major groove, making it difficult for proteins to make sequence-specific interactions. But any structural disruption, even a single mismatch, typically opens the major groove and facilitates sequence-specific interactions. While there are more options for sequence-specific interactions in the major groove, and such interactions have been observed with dsRBDs (Ryter and Schultz 1998), structural studies with the dsRBDs of ADARs show that widening of the minor groove correlates with a sequence-specific minor groove interaction involving the 2-amino group of guanosine (Stefl et al. 2010). Thus, it is possible that structural disruptions promote sequence-specific interactions of the dsRBDs of ADARs in either the major or minor groove.

Fascinatingly, it is observed that editing at an AU base pair, which creates an IU mismatch and thus another structural disruption, propagates the effect by also enhancing editing at a distance, leading to a reiterative model for ADAR editing (Uzonyi et al. 2021; Zambrano-Mila et al. 2023). Not surprisingly, editing of A:C mismatches, which restore base-pairing, do not propagate additional editing sites.

### But are there accessory factors?

While all studies to date indicate that nearest-neighbor preferences and selectivity are an intrinsic property of ADARs and the structures of their substrates, and do not require the participation of accessory factors, ADARs are indeed regulated (Deffit and Hundley 2016; Vesely and Jantsch 2021). ADARs are regulated at the transcriptional level, and this is particularly important for ADAR1, which in humans has four promoters, three constitutive and one interferon-inducible (Pfaller et al. 2021). Posttranslational modifications of the ADAR enzymes themselves can alter their activity, and as discussed in more detail below, localization of ADARs to the nucleus or cytoplasm plays a large role in which dsRNAs are edited (Vesely and Jantsch 2021); indeed, examples of factors that affect localization exist (Deffit and Hundley 2016). Finally, and importantly, the story is not over yet, and recent genome-wide and proximity-labeling experiments reveal candidate accessory factors (Hong et al. 2018; Quinones-Valdez et al. 2019; Freund et al. 2020; Cottrell et al. 2023). While identifying a number of interesting proteins whose effects on ADARs are yet to be explored, these studies also emphasize that other dsRBPs, by competing for binding to ADAR's dsRNA substrates, can regulate levels of editing. Such competition is a common theme in the regulation of dsRBPs (Cottrell et al. 2024).

### What are the immunogenic dsRNAs?

The observation in early studies of ∼50% A-to-I conversion in viral transcripts, and the completely base-paired dsRNA used for in vitro studies (Bass 1997), prompted some to question the biological relevance of ADARs. How in the world could something so promiscuous have a precise biological function? Many breathed a sigh of relief when the first recoding editing event was observed (Sommer et al. 1991). Most recoding events occur with remarkable precision, due to the fact that the large part of the dsRNA structure is encompassed by intronic sequences, allowing the base-paired regions that encompass coding sequences to be short, and with structural disruptions, very selectively edited (Bass 2002). In hindsight of course, the intrinsic, promiscuous activity should not have been ignored since we now know this pointed to one of the most important functions of ADARs: the marking of endogenous “self” dsRNA so that it will not trigger an aberrant immune response. This function has now been shown in mice and human cells (Hartner et al. 2004; Mannion et al. 2014; Liddicoat et al. 2015; Pestal et al. 2015), *Caenorhabditis elegans* (Reich et al. 2018) and *Drosophila melanogaster* (Deng et al. 2020). In mammals, the lack of ADAR editing has been shown to trigger an immune response through the MDA5 pathway, and consistent with this, the aberrant immune response observed in the absence of ADARs can be rescued by also knocking out MDA5 or MAVS (Quin et al. 2021).

A huge caveat is that as yet we do not know the immunogenic dsRNAs that lead to an aberrant innate immune response in the absence of ADARs. Because the vast majority of ADAR editing sites in mammals occur within inverted SINE elements (Alus in primates; B2 in rodents), it has been assumed these are the dsRNAs that must be kept at bay. In truth, this is far from proven. A recent review discusses the caveats associated with this hypothesis, and possible explanations, chief among these that the culprits might be dsRNAs that are harder to find, maybe because they are completely base-paired and/or heavily edited (Levanon et al. 2024).

Excitingly, a great deal of progress has been made recently in narrowing down the candidates, at least in mammals, and it seems likely that soon we will understand which dsRNAs trigger MDA5 when ADARs are knocked out. Thanks to thorough and in-depth studies, we know that it is the interferon-inducible ADAR1p150 isoform that is responsible for the editing that precludes an MDA5-induced interferon response (Pestal et al. 2015; Kim et al. 2021), and it is by focusing on edits specific to ADAR1p150, or RNAs that uniquely bind to p150, that the candidates have been narrowed (Sun et al. 2022; Kleinova et al. 2023).

Because the innate immune sensor MDA5 is in the cytoplasm, waiting for its viral dsRNA triggers, it has always been clear that immunogenic dsRNA must be in the cytoplasm at least some of the time. However, it has been presumed that edits that occur in the nucleus would protect self-dsRNAs once they entered the cytoplasm. Recent data suggest this is not the case, and it is editing in the cytoplasm that is important for keeping the immune response at bay. Indeed, the key feature of ADAR1p150's ability to protect self-dsRNAs is largely its cytoplasmic localization. Manipulations that cause the ADAR1p110 isoform or ADAR2 to localize to the cytoplasm can also prevent an MDA5-mediated interferon response (Sun et al. 2022; Kleinova et al. 2023).

While it is still the case that the majority of ADAR edits in mammals are in inverted SINE elements, the edits that are unique to ADAR1p150, and thus constitute the immunogenic dsRNAs, are largely in 3′ UTRs and nonrepetitive hyperedited sequences (Li et al. 2022; Sun et al. 2022; Kleinova et al. 2023). Some recent studies provide support for the idea that a subset of immunogenic dsRNAs arise from converging and diverging transcription to form *cis*-natural antisense transcripts (*cis*-NATs; Li et al. 2022; Sun et al. 2022). Since the more stable and completely base-paired a dsRNA is, the greater the fraction of its adenosines that will be edited, the categorization of an immunogenic RNA as hyperedited suggests that it is a highly base-paired dsRNA. By definition, the pairing of a sense RNA with its complementary antisense strand, as occurs in a *cis*-NAT dsRNA, creates a completely base-paired intermolecular dsRNA that would be highly edited. This raises an important caution when analyzing a potential intermolecular interaction. The amount of dsRNA that can be formed will be dictated by the strand that is lowest in abundance. In the presence of the complementary strand, the probability that the low abundance strand will exist in dsRNA is high, and the fraction of its adenosines edited will also be high. In contrast, the large majority of the most abundant strand will be present as a free single strand and will remain unedited. To ensure ADAR editing within an intermolecular dsRNA is detected, it is important to always include an analysis of editing in the strand of lowest abundance.

For the *cis*-NATs that are candidates for intermolecular ADAR targets because their 3′ ends overlap, that is, converging transcripts (Wright et al. 2022), it is interesting to consider known regulatory pathways that could alter the overlap, such as alternative splicing or polyadenylation. In this regard, a recent paper implicating elongated 3′ UTRs as important for an MDA5-mediated interferon response in neuronal cells may also provide a clue (Dorrity et al. 2023).

### My favorite mysteries

While it seems we may know the immunogenic dsRNAs soon, more mysteries await. In mammals and *C. elegans*, most ADAR editing occurs in noncoding sequences, and editing in codons is very rare (Reich and Bass 2019). In contrast, recoding events are more common in *D. melanogaster* (Yu et al. 2016; Zhang et al. 2017), and in coleoid cephalopods (octopuses, squids, cuttlefishes), recoding sites are very abundant, with analyses of squid showing recoding editing events in ∼60% of all mRNAs, with many transcripts having multiple sites (Alon et al. 2015). The amazingly promiscuous recoding editing in cephalopods has been nicely correlated with fascinating phenotypes (Birk et al. 2023; Rangan and Reck-Peterson 2023). However, as yet, the structures that presumably mediate editing in cephalopods are unknown. Until structures are identified, it remains possible that dsRNA is not involved. However, the intriguing temperature-dependent editing changes reported in both of these recent studies support the idea of dsRNA-mediated editing. As poikilotherms, neither *D. melanogaster* nor cephalopods can regulate their internal temperature, except by changing location, and indeed, studies show that editing increases when these animals are in colder temperatures (Rieder et al. 2015; Buchumenski et al. 2017; Birk et al. 2023). Since the thermodynamic stability of dsRNA increases as the temperature is lowered, this is entirely consistent with prior discussions in this article of studies correlating more editing sites in more stable ADAR dsRNA substrates.

Finally, there are many additional questions about the interplay of ADAR editing and the antiviral response that are as yet unanswered. Since most RNA viruses replicate in the cytoplasm, the simple idea that ADAR editing marks dsRNAs as “self” seemed logical when it was thought that the protective editing occurred in the nucleus. Now that recent studies indicate that protective editing occurs in the cytoplasm, the question arises as to how “nonself” viral dsRNA is protected from editing in the cytoplasm. Of course, viral transcripts were among the first discovered ADAR substrates (Bass 1997), so in truth, there have always been loopholes in this idea. Indeed, viral replication often occurs in virus-induced membrane-bound mini-organelles (Nishikiori et al. 2022), and it will be important to understand ways that viral dsRNA might encounter ADARs in the cytoplasm. In this regard, it will be important to carefully monitor temporal aspects of ADAR editing of endogenous transcripts, and how levels of ADARs change, in particular the interferon-inducible ADAR1p150, when viral dsRNA starts to increase after infection. A recent analysis of RIG-I activities during a viral infection emphasizes the importance of careful studies over time (Thoresen et al. 2023).

### Going forward: the NASEM report

As exemplified in this special issue of *RNA* devoted to *Epitranscriptomics*, understanding of the pathways and factors that lead to RNA modifications, and their biological functions, has increased exponentially in recent years. Certainly, the time for epitranscriptomics is now. That is the consensus in the recently released National Academies of Sciences, Engineering, and Medicine (NASEM) report, *Charting a Future for Sequencing RNA and Its Modifications: A New Era for Biology and Medicine*. This report resulted from the year-long deliberations of a NASEM committee that I was honored to co-chair with Taekjip Ha. As highlighted by the title of the report, the committee was tasked with evaluating the needs for achieving direct sequencing of RNA and its modifications; in other words, should we determine the epitranscriptome? There are obvious analogies to such an effort and the Human Genome Project, but determining an epitranscriptome is quite a different task from determining a genome. While an individual might be reasonably well described by one genome sequence, many different epitranscriptomes would be needed to describe the diverse transcripts unique to different tissues and cells of the individual, and even these will change with the environment. Thus, as detailed in the report, the goal was developing a plan to enable technology that would allow anyone to determine the epitranscriptome of their choice. It was an amazingly knowledgeable and conscientious committee, and the editors of this special *RNA* issue, Kate Meyer and Tao Pan, were key members of the committee, and at the start of this volume provide more details of the final report. After learning so much from the research of the NASEM committee, it is my opinion that we should try. We should put large resources into efforts that will enable any of us to sequence our favorite epitranscriptome. It is an exciting time that certainly I would never have imagined when I left the movie theater to image my TLC plates.
